# Positive feedbacks in deep-time transitions of human populations

**DOI:** 10.1098/rstb.2022.0256

**Published:** 2024-01-01

**Authors:** Mauricio Lima, Eugenia M. Gayo, Sergio A. Estay, Andone Gurruchaga, Erick Robinson, Jacob Freeman, Claudio Latorre, Darcy Bird

**Affiliations:** ^1^ Facultad de Ciencias Biológicas, Pontificia Universidad Católica de Chile, Santiago, RM 8320000, Chile; ^2^ Center of Applied Ecology and Sustainability (CAPES), Pontificia Universidad Católica de Chile, Santiago, RM 8320000, Chile; ^3^ Departamento de Geografía, Pontificia Universidad Católica de Chile, Santiago, RM 8320000, Chile; ^4^ Instituto de Ciencias Ambientales y Evolutivas, Universidad Austral de Chile, Valdivia 5090000, Chile; ^5^ School of Human Evolution and Social Change, Arizona State University, Tempe, AZ, 852879, USA; ^6^ Native Environment Solutions LLC, Boise, ID, 83250, USA; ^7^ Anthropology Program, Utah State University, Logan, UT, 84322, USA; ^8^ The Ecology Center, Utah State University, Logan, UT, 84322, USA; ^9^ Department of Anthropology, Washington State University, Pullman, 99164, USA

**Keywords:** human expansions, population, cooperation, positive feedback

## Abstract

Abrupt and rapid changes in human societies are among the most exciting population phenomena. Human populations tend to show rapid expansions from low to high population density along with increased social complexity in just a few generations. Such demographic transitions appear as a remarkable feature of *Homo sapiens* population dynamics, most likely fuelled by the ability to accumulate cultural/technological innovations that actively modify their environment. We are especially interested in establishing if the demographic transitions of pre-historic populations show the same dynamic signature of the Industrial Revolution transition (a positive relationship between population growth rates and size). Our results show that population growth patterns across different pre-historic societies were similar to those observed during the Industrial Revolution in developed western societies. These features, which appear to have been operating during most of our recent demographic history from hunter–gatherers to modern industrial societies, imply that the dynamics of cooperation underlay sudden population transitions in human societies.

This article is part of the theme issue ‘Evolution and sustainability: gathering the strands for an Anthropocene synthesis’.

## 1. Background

Abrupt and rapid changes in the size and complexity of *Homo sapiens* populations are among the most exciting socio-ecological phenomena. Human populations tend to show rapid expansions from low to high population density and social complexity in relatively few generations [1–3]. Such demographic transitions appear as a remarkable feature of *H. sapiens*' population dynamics, most likely fuelled by the interaction of cultural/technological innovations that actively modify the environment and changes in life history in response to environmental and cultural changes [4–10]. In fact, the proposed Anthropocene Epoch can be viewed as a global process of human niche construction driven by the synergic interaction between population dynamics and the dynamics of cultural evolution [11,12]. Most of our recent demographic history, from pre-historic hunter–gathers to agrarian to modern industrialized societies, can be represented by a similar structural cooperative dynamic, the signature of human population transitions. We find it exciting and essential to perceive that the Anthropocene Epoch has been caused by this same process but powered by a new rich source of energy, i.e. fossil fuels.

Several authors have noticed the correspondence between population growth events and significant social–technological changes [1,13–16]. People create technology that extracts more energy from local ecosystems that sustains more people, and, in turn, populations produce more effective technologies for extracting resources, triggering a positive feedback between technological change and population growth [10,12,17–24].

For example, an important process in the population growth of *H. sapiens* was the development and adoption of agriculture, widely labelled the Neolithic demographic transition (NDT) [25,26]. Human populations experienced a rapid increase in density as farming groups began displacing foragers about 10 000–11 000 years ago from the Levant in the Middle East through a series of expansive demographic pulses [27]. In general, NDT studies have focused on describing the underlying demographic mechanisms [25] and the spatial expansion processes of agricultural societies [27,28]. Most studies of the NDT assume that the population expansion process was caused by either exponential or logistic growth initiated by the adoption and spread of agriculture [29,30]. However, several studies indicate that human foragers also experience demographic transitions [31], and, over thousands of years, some foraging and farming populations experience similar rates of exponential expansion [32]. These studies suggest that the NDT represents one of potentially many different positive feedback relationships between population growth and technological change that have occurred throughout the deep history of *H. sapiens* on earth.

Another example of a human population transition was the population boom exhibited by *H. sapiens* following the Industrial Revolution [13,33,34]. A key, but usually overlooked, demographic feature of the Industrial Revolution is the positive relationship observed between population growth rates and population size over approximately 300 years during this revolution (1650–1970) [35]. This structural pattern of population growth represents the cooperation demographic principle of population dynamic theory (PDT) [36]. During the Industrial Revolution, new cooperative social institutions, such as public stock exchanges, capital accumulation and open markets drove technological innovations. Fossil fuels allowed western societies to extract natural resources at intensities and magnitudes never before experienced. The positive feedback among growth rates and population size interacted with the new technologies for enhancing the energy gradient transferred from ecosystems to human systems. Similar to the NDT, the Industrial Revolution, and the demographic changes associated with it, are often treated as unique processes that delineate modern from pre-modern human societies (e.g. [37]). However, it is unclear whether the positive feedback between population size and growth rates between 1650 and 1970 was unique or typical of demographic transitions among *H. sapiens* throughout our species history.

The general objective of this study is to connect Anthro-Ecological theory (AET), in particular the idea that there is a positive feedback among niche construction, ecosystem engineering and human population size [11,22,38], with the analytical approach of PDT [36,39,40]. AET builds on previous theoretical studies that describe the coupled population-culture dynamic to explain sudden demographic transitions in human populations [5,41–47]. The basic tenet is that technological innovations modify the environment and the energy fluxes from the environment, which affects human population growth. We propose that the demographic transitions of pre-historic human populations are characterized by a positive relationship between population growth rates and size, as a dynamic signature of the underlying positive feedback loop between the increase in population sizes and the improvements in resource extraction via cultural evolution and technological innovations [38]. Even when the positive feedback owing to cooperation (i.e. Allee-type demographic dynamics) have been detected in pre-industrial societies [48,49], to the best of our knowledge, no study has addressed the population growth processes behind the sudden demographic transitions of human societies or the potential generality of such transitions.

## 2. Material and methods

We used the cleaned and scrubbed p3k14c global radiocarbon database [50], which is the most comprehensive dataset of curated and georeferenced archeological ^14^C data on earth. We sampled the radiocarbon ages using the 17 ArchaeoGlobe World Regional analytical units and pooled the ages by those geographical units [51]. We focused on *ArchaeoGLOBE* regions as their record of archeological ^14^C-dates is more statistically robust [50]. Likewise, data quality/availability for past changes in socio-cultural phases has been critically assessed [52,53]. Any radiocarbon date that was not located within a given region (common for archaeological sites located on land spits, sand bars, generally close to water, etc.) was assigned to the closest ArchaeoGLOBE region. These ArchaeoGLOBE regions are divided into World Regions in the dataset (e.g. Eastern Africa, Middle and Southern Africa, Central Asia). We produce summed probability distributions (SPDs) of regional radiocarbon dates at the ArchaeoGLOBE regional level and analyse these data at the World Regional Level [51].

To produce the SPDs, we first examined the radiocarbon ages per unit area and histograms of uncalibrated radiocarbon ages to identify regions where research bias might limit our ability to interpret the SPDs. For example, regions like Southern Asia have many ages from between 8 000 and 4 000 cal BP and none before or after. We suspect that this is owing to research bias and the availability of data for inclusion in the p3k14c dataset. After noting potentially suspect cases where we might detect false demographic transitions owing to research and data inclusion biases, we computed unnormalized SPDs for each of the 17 ArchaeoGlobe World Regions from archeological ^14^C-dates to generate regional time series for past population sizes.

We used the *rcarbon* package [54] in R to develop SPDs of radiocarbon ages. Recent work comparing archaeological radiocarbon time series to other independent population proxies has illustrated the usefulness of this proxy to reconstruct deep time population dynamics [55]. We calibrated the archaeological radiocarbon ages (rcarbon::calibrate) by using IntCal20 [56] and SHCal20 [57] curves for radiocarbon dates from the North and South Hemisphere, respectively. We produced unnormalized SPDs (rcarbon::spd) for the past 15 000 years, and we used a 200-year rolling average to smooth over extreme variation in the SPDs. Next, we trimmed the edges off of each SPD to avoid misleading values of 0 owing to no data. Finally, to fit PDT models, we used the smoothed 200-year rolling SPD data and summed each of the annual SPD values at time-step intervals of 30 years. We did this because our models are specified to capture population trends over a generational interval of 30 years. Summing is one way to capture large population trends and avoid intra-generational variability.

**Figure 1.**
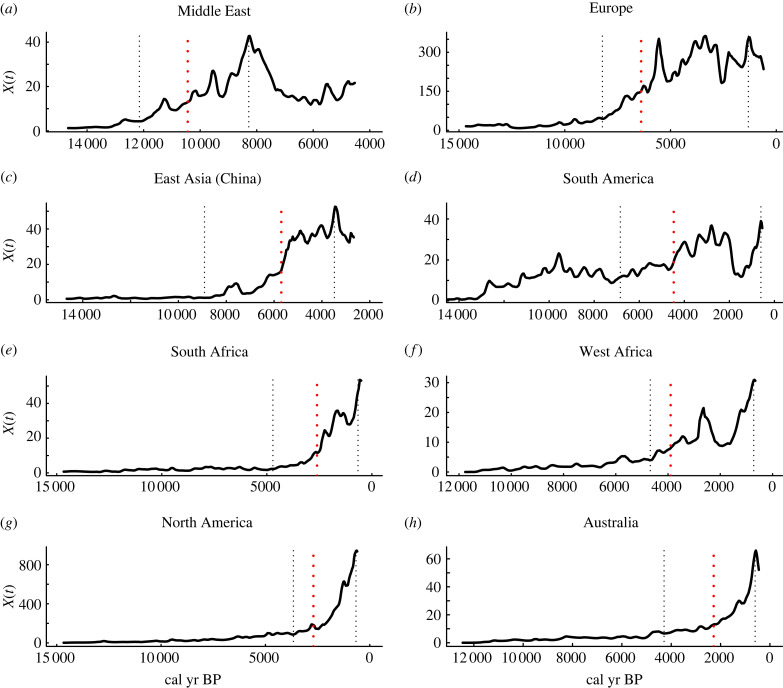
Regional SPD time series for different geographical regions (*y*-axis) and the calibrated years before the present (*x*-axis); the vertical dotted red line denotes the estimated breaking date of the major shifts in population expansions, and the vertical black dotted lines the periods of population growth used in the population transitions analyses. (*a*) Middle East; (*b*) Europe; (*c*) East Asia (China); (*d*) South America; (*e*) South Africa; (*f*) West Africa; (*g*) North America and (*h*) Australia. Periods of major demographic transitions in each SPD time series were estimated by using the least squares method, so pre-shift and post-shifts means SPD values were estimated concurrently with the change point (electronic supplementary material, figure S1).

Of the 17 ArchaeoGlobe World Regions, we chose eight for analysis. We chose these eight cases because they have the least suspected research and data inclusion bias, and we wanted to represent each of the six pre-historically inhabited continents. In practice, SPDs represent an indirect estimate of population levels that can, but have yet to be, explained on archeological [58–61] or ecological principles [62–64]. Our goal here is to establish a formal theoretical foundation for explaining the deep history of human population growth. Our empirical analysis is an exploratory demonstration of the usefulness of dynamic population theory. As we note later in the discussion, more work at smaller spatial scales and integrated with data on changes in subsistence and social organization is an important direction for future research that our study warrants. In summary, we study eight cases (figure 1; electronic supplementary material, table S1) that may exhibit demographic trends and sudden population expansions including pre-historic systems from different bioclimates, including Europe, the Near East, North Africa, East Asia (mostly China), South America (mostly Central Andes Area), South Africa, North America and Australia.

### (a) Diagnostic tools for summed probability distribution time series

A diagnosis is critical when dealing with complex systems where it is impossible to obtain detailed quantitative information, such as pre-historic human populations. In the study of population dynamics through time series analysis, the art of prior diagnosis is a fundamental step to recognize the type of dynamics exhibited and detect the determining factors [36,39,40]. We applied these techniques to SPDs using the following steps.

First, we plotted the SPD time series to check for non-stationarity in the mean SPD values and the variance [39]. Plotting the raw SPD time series is important because discontinuities and trends are often apparent from a preliminary examination of the trajectories through time. Second, we used a key tool of population dynamic analysis: the phase portrait. The phase portrait is obtained by plotting the data in the log_e_ (SPD) – log*_e_* (reproductive rate = *R_t_* = log*_e_* SPD*_t_*_+ 1_ − log*_e_* SPD*_t_*) phase space [36]. The phase portrait provides useful information on whether shifts occurred in the SPD trajectories. By examining the shape of the curve in a phase portrait, we can diagnose what kind of population dynamics predominate and detect discontinuities or sudden shifts in the time series from low-density to high-density dynamics and vice versa. Similarly, these kinds of plots are essential to determine if sudden transitions from low to high population sizes/densities are driven by exponential (constant rate growth) or hyper-exponential (accelerated growth) population growth rates.

Demographic transitions can be characterized by hyper-exponential (accelerated) population growth rates in the long run. In this case, we first expect accelerated growth rates as population size increases (i.e. positive feedback owing to cooperation), but then a decrease in growth rates as population levels increase (i.e. negative feedback owing to competition). This humped relationship fits the quintessential western European demographic pattern after the Industrial Revolution [35]. If this model applies to past human societies, then we would expect a pattern of accelerated growth rates as population size increases during each demographic transition event.

Finally, we determined periods of major demographic transitions in each SPD time series by applying a statistical procedure borrowed from econometrics to detect structural changes in a linear process. We tested deviations from stability in a classical linear regression model of SPD against time assuming that there is one unknown shift point in the SPD series in time. Basically, we tested the null hypothesis of no structural changes, the regression coefficient is the same during the entire time interval, against the alternative hypothesis that the regression coefficient shift from one stable regression relationship to a different one. Thus, there are two segments in which the regression coefficients are constant. These change points were estimated by using the least squares method, so pre-shift and post-shifts means SPD values were estimated concurrently with the change point [65]. We fitted for each SPD time series the linear regression model, assuming one breakpoint by least squares methods using the *strucchange* library in the R platform (scripts and data fully available). After selecting and splitting each SPD time series for the major demographic transition periods, we fitted population growth models to these SPD sequences.

### (b) Population growth models and statistical analyses

We begin with a simple, non-structured, discrete-time theoretical model describing the effects of intra-specific competition on the dynamics of a population growing in a finite environment [39,40];

2.1 $$x_{t+1}=x_{t}\cdot r_{m}\cdot e^{\left[-s\cdot (1-k)\cdot x_{t}\right]},$$

where *x_t+_*_1_ is the population size at time *t* + 1, *r_m_* is the (mean) potential reproductive rates of the individuals (when they have no competitors and resources are fully available), *s* is a positive constant representing the individual resource requirements, and *k* is a positive constant 0 < *k* < 1 that reduces the potential reproductive rates each time an additional individual is added to the population; the lower the *k* value the faster the decrease in reproductive rates [39,40]. The model describing the effects intra-population cooperation can be represented as the opposite force of equation (2.1) using the same ecological base the discrete-time step model of cooperation is

2.2 $$x_{t+1}=x_{t}\cdot r_{m}\cdot e^{\left[-z\cdot (1-k^{\prime })\cdot (1/x_{t})\right]},$$

where *r_m_* is the mean net reproduction rate, *z* is a positive constant that represent effect that some environmental hazards impose on the individuals of the population. For example, when some important resources have defences, or some environmental factor represents a threat. Therefore, in this case, and in opposition to equation (2.1), *k’* is a positive constant 0 < *k’* *<* 1 that increases the potential reproductive rates towards the maximum reproductive rate *r_m_*, each time an additional individual is added to the population. The lower the *k′* value, the faster the reproductive rate increases. In fact, both the expressions, *s*(*1 − k*) in equation (2.1) and *z(1 − k′)* in equation (2.2) can be written as a simple constant *c* [40] and *w*, being the intensity of competition and the amount of cooperation needed to overcome some environmental hazards, respectively. The higher the *c* value the faster the decrease in population growth rates with population density. Otherwise, the higher the *w* value the higher the population size needed to increase population growth rates. Because the net rate of change from generation *t* to *t* + 1 is measured as the ratio *x_t + 1_/x_t_* = *r_t_*, equations (2.1) and (2.2) can be combined in a single model including the effects of both terms, competition, and cooperation as

2.3 $$r_{t}=r_{m}\cdot e^{\left[-c\cdot x_{t}-w\cdot (1/x_{t})\right]}.$$

For analytical convenience, we write equation (2.3) in terms of the logarithmic (*per capita*) reproductive rate log_e_ (*r_t_*) = *R_t,_* log_e_ (*r_m_*) = *R_m_* and log_e_ (*x_t_*) = *X_t_* as

2.4 $$R_{t}=R_{m}-c\cdot e^{\left(X_{t}\right)}-w\cdot e^{\left(-X_{t}\right)}.$$

The shape of the reproductive curve *R-X* is determined only in terms of these three parameters: *R_m_* (the logarithmic (mean) maximum reproductive rate), *c* (intensity of the intra-population competition) and *w* (intensity of cooperation), which all have a clear ecological and population dynamic interpretation.

We determined the temporal sequences of the SPDs time series characterized by population growth transitions by drawing the logarithmic reproductive rates time series (*R_t_*) and the *R-X* reproductive curves [39,40]. Finally, to each time series sequence we fitted equation (2.4) through nonlinear least squares using the *nls* (nonlinear least squares) library in the R platform (scripts and data fully available).

## 3. Results

### (a) Population transitions

Our cross-continental analysis suggests that sudden demographic transitions in past human populations occurred recurrently and transversely during the last 14 000 cal yr BP (figure 1). For example, the Middle East SPD time series suggests a significant breaking point (i.e. population shift) around approximately 10 440 cal yr BP, but the increasing trend started to differentiate from the basal SPD level around approximately 12 000 cal yr BP. This implies that the expansion phase over the Middle East lasted several thousand years (figure 1*a*; electronic supplementary material, figure S1*a*). A major structural change (significant breaking point) is evident for Europe at approximately 6390 cal yr BP. Still, the European SPD time series suggests that the demographic boom started by approximately 8200 cal yr BP, and then fluctuated largely for over 3500 years (figure 1*b*; electronic supplementary material, figure S1*b*). In the case of East Asia, the major significant population shift occurred by approximately 5700 cal yr BP, differentiating from the basal line around 8000 cal yr BP (figure 1*c*; electronic supplementary material, figure S1*c*). The significant shifting population transition in South America occurred at approximately 4500 cal yr BP, but this trend was apparent since approximately 7200–6800 cal yr BP (figure 1*d*; electronic supplementary material, figure S1*d*). The SPD time series for South Africa attests to an abrupt and significant shift by approximately 2600 cal yr BP, which started to increase from the basal regression line around approximately 4500 cal yr BP (figure 1*e*; electronic supplementary material, figure S1*e*). The SPD time series from West Africa indicates a significant population shift at approximately 3900 cal yr BP with an increasing pattern starting at approximately 6600 cal yr BP (figure 1*f*; electronic supplementary material, figure S1*f*). North American populations experienced a significant shift (breaking point) at approximately 2700 cal yr BP that starts around approximately 5100 cal yr BP (figure 1*g*; electronic supplementary material, figure S1*g*). The significant population shift in Australia arose at approximately 2300 cal yr BP with an expansive process starting since 4300 cal yr BP (figure 1*h*; electronic supplementary material, figure S1*h*).

**Figure 2.**
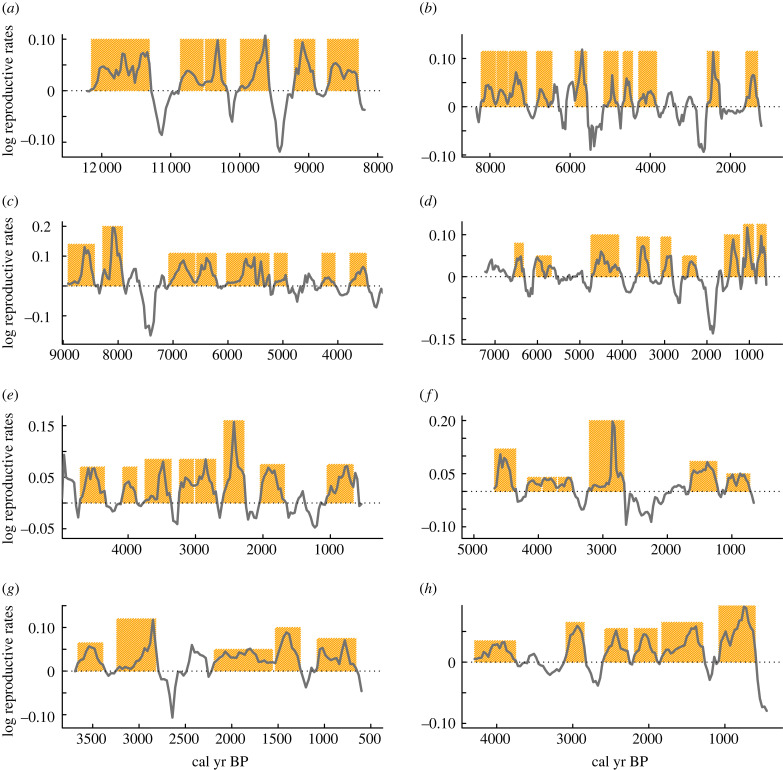
Time series of the logarithmic net reproductive rates (*R_t_*) based on the selected periods of sustained growth from the SPD time series. (*a*) Near East, (*b*) Europe, (*c*) East Asia (China), (*d*) South America, (*e*) South Africa, (*f*) West Africa, (*g*) North America, and (*h*) Australia. The shaded orange areas denote each of the sequences of uninterrupted periods of population growth used for fitting the model of equation (2.4) (see Material and methods for details).

The time series of the logarithmic reproductive rates (*R_t_*) during periods of population growth exhibited a ‘wave-like’ pattern of accelerated/de-accelerated rates (figure 2*a–h*). The time series plot of population growth rates from the Middle East shows eight waves of demographic expansions between approximately 12 000 and 8000 cal yr BP (figure 2*a*). The human population expansion occurred in Europe during the last 8000 years seems to be characterized by 10 waves of increasing population growth rates (figure 2*b*). In the same vein, the human population expansion in East Asia was characterized by eight population growth waves, which occurred between approximately 8500 and 3400 cal yr BP (figure 2*c*). The demographic expansion observed in South America during the period approximately 7000–600 cal yr BP exhibited nine waves of population growth rates (figure 2*d*). Over the past 7000 years, human population growth in West and South Africa displays seven and eight population waves of growth, respectively (figure 2*e,f*). In North America, the process of human demographic expansion during the last 3700 years displays five population growth waves (figure 2*g*), while in Australia, human expansion during the last approximately 4300 years showed five waves of population growth (figure 2*h*).

**Figure 3.**
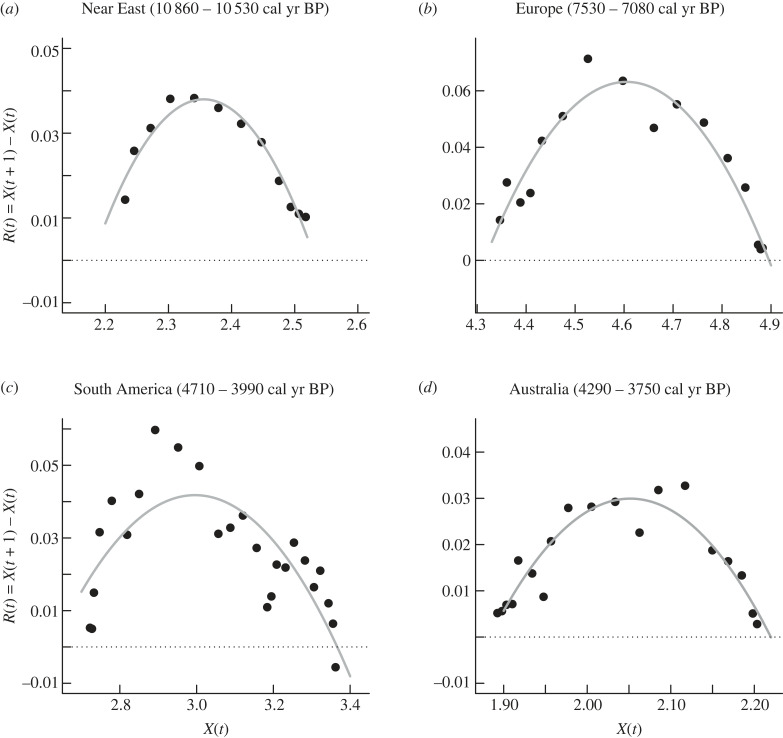
Examples of the observed reproduction curves (*R_t_* = (*X_t+1_* − *X_t_)* against (*X_t_*) of the SPD times sequenced time series when logarithmic reproduction rates are plotted against the natural logarithm of SPD (*X_t_*) with a time lag of one generation (30 years). (*a*) Near East, (*b*) Europe, (*c*) South America, and (*d*) Australia. The fitted curves (model 5) parameters are shown in the electronic supplementary material, table S1.

**Figure 4.**
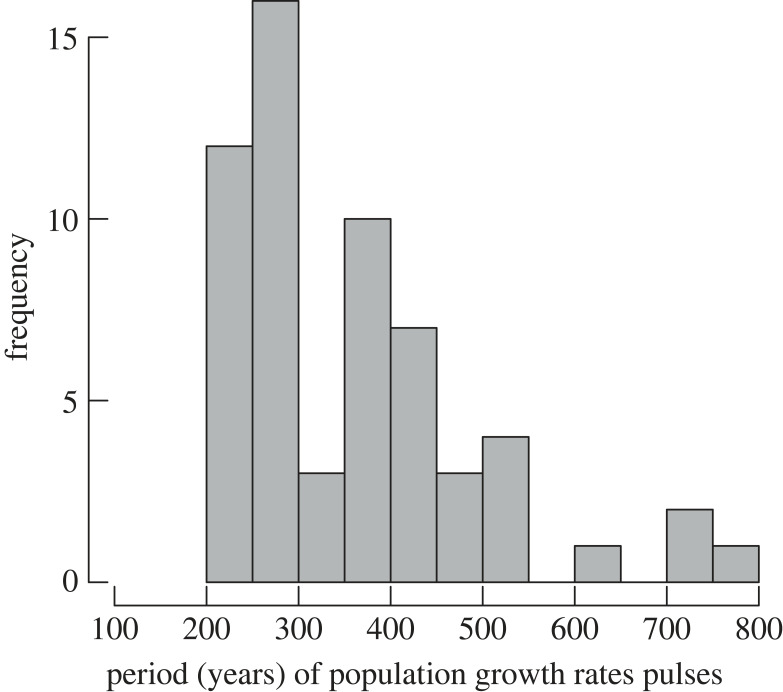
Frequency distribution of the periods (years) of uninterrupted population growth observed during the demographic transitions (mean = 365 years).

The phase portraits revealed that almost all demographic transitions were characterized by humped ‘waves’ of growth rates with a positive/negative relationship with the logarithmic SPD values (figure 3; electronic supplementary material, figure S2 and table S1). Importantly, this pattern occurs independently of the subsistence strategy, affecting both hunters–gathers and agrarian societies. Overall, population growth pulses/waves showed an average duration of 365 years, and most population expansions lasted between 250 and 450 years (figure 4). The cooperation/competition model (equation (2.4)) indeed fit well with population growth waves on all continents. For instance, five of six demographic expansions observed in the SPD time series from the Near East (electronic supplementary material, figure S3 and table S1) are best explained by this mechanism. The same is true for every wave of population growth described in the SPD time series from Europe and East Asia (electronic supplementary material, figures S4 and S5, table S1). The nine population growth waves observed in South America SPD time series were quite well described by the model from equation (2.4) (electronic supplementary material, figure S6 and table S1), while four of the five population growth rate pulses from North America SPD time series follow the same humped pattern (electronic supplementary material, figure S7 and table S1). All the population growth waves observed for West and South Africa, as well as in Australia were well fit by a cooperation/competition population model (electronic supplementary material, figures S8–S10 and table S1).

## 4. Discussion

By implementing a theoretical/empirical modelling approach from the PDT, here we provide empirical evidence that the long-term dynamics of past human populations appears tied to multiple demographic transitions. Specifically, these results illustrate how Allee-type population growth processes—i.e. positive feedback owing to cooperation—was an inherent phenomenon in the trajectory of human populations, at least during the Holocene. These results support earlier work that attempted to explain how exponential growth could occur in hunter–gatherer populations [48]. Thus, neither the NDT, nor the western European demographic trajectory after the mid-eighteenth century, are unique but, rather, specific instances of a general pattern of human population growth. This means that the demographic impacts that occurred during the Industrial Revolution have deeper time precedents. We suggest that such demographic transitions are qualitatively and mechanistically comparable, and governed by the positive feedback between population sizes and the improvements in resource extraction/use via cultural or/and technological innovations. These results reveal a much more complex process explaining how humans evolved from small populations of a few thousand individuals during the Pleistocene to approximately 8 billion people, becoming the major driving force of the Earth System.

The impact of positive feedbacks or Allee effects on animal population dynamics has been recognized for decades [36,66,67]. In fact, numerous studies describe the mechanisms involved in generating positive feedbacks across animal taxa [68]. However, in human populations the role of such positive feedbacks has been little studied [48], despite a rich base of available conceptual tools to explain them, such as, niche construction theory, cultural evolution, cultural cycles and AET [3,11,69–71]. Lima & Berryman [35] found evidence for Allee-type dynamics in the trajectory of the global human population during the Medieval Age and the Industrial Revolution, both triggered by the reciprocal relationship between population sizes, food production and rate of technological innovations. Similarly, Gayo *et al*. [49] qualitatively identify a positive feedback loop between demographic levels, ecosystem engineering, technological innovations and cooperation practices in the Atacama Desert after the agriculture adoption (approx. 3300 cal yr BP). Abrupt accelerated population increases are evident in the trajectory for other pre-industrial societies, but these are usually interpreted as the result of exponential growth after the agriculture spread over western Europe [72,73], Eurasia [26], Near East [74] as well as over North [75] and South America [30,49].

The Agricultural or Neolithic Revolution represents an important socio-economic change in pre-historic societies that led to increased availability of food per unit [76]. Because Neolithization was not a spatially widespread phenomenon, then positive feedbacks should only emerge as a pattern—i.e. circumscribed—to regions where societies developed agriculture. Our results, however, contradict this corollary, and support earlier work [48,62]. Precisely, we document that sudden and rapid changes from a state of equilibrium (relatively constant population size) to one of demographic expansion (population growth) were recurrent across all continents, and under different bioclimates, social complexity levels and economic systems (e.g. hunter–gatherers, agrarian). In line with previous studies [1,2], the consistent pattern reproduced in this cross-continental study indicates that one of the most remarkable features of the long-term dynamics of human populations are demographic expansions. Actually, some studies have suggested the importance of a positive feedback among population size/density and the dynamics of cultural accumulation in pre-historic hunter–gatherers [6,17–19] as well as the Allee-benefits of population aggregation over a small region where societies never adopted agriculture or urbanization [48].

In all cases studies here, demographic transitions appear fuelled by the ability to accumulate cultural/technological innovations (see the electronic supplementary material, S1). The existence of such a demo-cultural dynamic is hardly new. Most theoretical models for human population dynamics acknowledge that demography and culture are intimately linked and often reinforce each other [5,42–46,77]. Nevertheless, a missing piece in the puzzle is how these forces interact in the long-term and explain the human–culture–environment relationship. In this sense, our empirical cross-cultural analysis based on the analytical approach of the PDT highlights the mutual positive feedbacks between population growth, niche construction processes and ecosystem engineering, which are mediated by cultural and technological accumulation [5,17–19].

Thus, we provide additional support for the notion that population, cooperation, and cultural trajectories are structured in a feedback relationship that impacts the evolution of the socio-cultural human niche [12,78,79]. According to models of niche cultural construction, humans of one generation ‘build’ new environments that are inherited by the following generation, which in turn modify it again (in the same direction) for the next generation [11,52,70,80]. This leads to a coupled dynamic between niche construction and ecological inheritance. Therefore, environmental modifications tend to improve the average living conditions of individuals, increasing population growth rates and population density [17,71]. The combination of innovation, social complexity, and ecosystem engineering capacity is an explosive ‘cocktail’ responsible for the human population expansion and the impact on the planet's surface [11,52,80,81]. Any innovation or cultural change that improves the access to new or more abundant resources impacts population size. For instance, in most case studies of agrarian societies reviewed here (see the electronic supplementary material, S1), demographic expansions occurred after the adoption of agrarian practices that involved the incorporation, adjustment and intensification of technologies as well as cooperative behaviours (e.g. domestication, land-use changes, urbanization, irrigation, metallurgy). Still, innovative hunting/foraging strategies also inevitably amplified the access to resources that were not previously available for pre-historic hunter–gatherers [48] (see the electronic supplementary material, S1). Such is the case of changes in the food production and hunting in Australia around 4200 cal yr BP [82] and the emergence of a new lithic tools over West Africa by 5000 cal yr BP [83]. Demographic expansions through the construction of socio-cultural niches necessarily requires that the cultural/technological accumulation increases more rapidly than population sizes [3,6,21,80,84,85]. So, under this loop as population size increases, the demographic and social environment favours a further expansion of cultural innovations. In this sense, our results agree with the idea that technological and cultural innovations seem to be more reliable in larger populations [18,21,86], which can fuel positive feedbacks in population growth rates.

Although this study indicates that human populations responded to cultural/technological innovations through relatively sudden equilibrium shifts throughout the Holocene, we also found that temporal trajectories tend to resemble a metastable equilibrium marked by thresholds at which ‘positive’ or ‘negative’ shifts of equilibrium level take place. This is consistent with the classical cooperation/competition principle of the PDT. In practice, it arises from the fact that population growth cannot go unchecked, even if new technological innovations spur the extraction of resources for further population growth, since increased pressures on available resources triggers intra-population competition [36]. This general process is observed in all of the phase portraits in this study, with different levels of intensity (the decreasing section of the curves) generating population equilibrium, stagnation, and even, in some cases, population collapses. In fact, all phase portraits studied here show cooperation and competition phases in agreement with the concepts of marginal returns in the level of social complexity of human institutions [87]. More importantly, most growth waves were characterized by periods of around approximately 300–400 years, reinforcing the idea that environmental limitation represents a key element for the positive feedback between technological change and population growth rates.

Demographic boom-and-bust cycles are documented in different Holocene societies (e.g. [72,74]). Climate change is usually investigated as a potential driver since favourable (adverse) conditions could enhance (reduce) resource availability. This exogenous factor, however, does not fully account for demographic transitions detected in Europe [72], the Near East [74] or the Atacama Desert [81]. Cumulative negative impacts from long-term ecosystem engineering, or other endogenous factors (social complexity, migration) have also been evoked to explain stagnation or collapse phases, but their role has thus far not been illustrated empirically. In this sense, our study offers an alternative mechanism for the phenomenon that affected populations with different socio-cultural, and bioclimate backgrounds, which cannot be explained by simple correlations or case-specific driven factors. That is, nothing about human demography, at least, during the last 12 000 years makes sense without considering the positive feedback relationship between population growth and cultural innovations.

## 5. Conclusion

Our results suggest that a much more flexible approach is required to understanding human population dynamics by including cooperation and competition as general principles driving demographic changes. Given the present controversy about how human population dynamics can be a cause or a consequence of socio-cultural innovations, we present support for the idea of a mutual feedback relationship between demography and cultural dynamics [11,17–21,23,73]. Pre-historic human societies exhibited the same humped reproduction curves during most of the population transitions as during the industrial revolution in developed western societies [35].

Over much of the Holocene, human population trajectories experienced waves of accelerated growth rates, probably driven by a positive feedback loop among population size and technological innovations, to a ‘propensity towards unsustainability and socio-ecological crisis' [38, p. 1096] and disruptive ‘upscaling and downscaling' processes’ [79]. We propose that the accelerated population growth of industrial societies was caused by the same process as most of the previous historical transitions, but powered with a new rich source of energy, i.e. fossil fuels. Moreover, these features appear to have been operating during most of our recent demographic history, from pre-historic hunter–gathers, through agrarian to modern industrialized societies. Thus, the structural instability of cooperation dynamics seems to be the signature of population transitions in human societies.

## Data Availability

We used the p3k14c global radiocarbon database, which is the most comprehensive dataset of curated and georeferenced archeological 14C data available from the Zenodo repository: https://zenodo.org/record/7877347#.ZEwqny-R1p [50]. The data are provided in the electronic supplementary material [88].
